# Establishing minimal clinically important difference for effectiveness of corrective exercises on craniovertebral and shoulder angles among students with forward head posture: a clinical trial study

**DOI:** 10.1186/s12887-022-03300-7

**Published:** 2022-04-27

**Authors:** Zahra Heydari, Rahman Sheikhhoseini, Shahnaz Shahrbanian, Hashem Piri

**Affiliations:** 1 Department of Corrective Exercise & Sport Injury, Faculty of Physical Education and Sport Sciences, Allameh Tabataba’i University, Tehran, Iran; 2 Department of Corrective Exercise & Sport Injury, Faculty of Physical Education and Sport Sciences, Allameh Tabataba’i University, Western Azadi sport complex boulevard, Hakim Highway, Tehran, Iran; 3 Department of Sport Science, Faculty of Humanities, Tarbiat Modares University, Tehran, Iran; 4 Department of Corrective Exercise & Sport Injury, Faculty of Physical Education and Sport Sciences, Allameh Tabataba’i University, Tehran, Iran

**Keywords:** Posture, Head, Shoulder, Exercise, Minimal clinically important difference

## Abstract

**Background:**

Previous studies have addressed the effects of different exercises and modalities on forward head posture (FHP), but the minimal clinically important difference (MCID) of the effect of exercises on FHP remains unclear. Therefore, this study aimed to investigate the effects of selective corrective exercises (SCEs) on the craniovertebral angle (CVA) and shoulder angle (SA) in students with FHP and to establish MCID for these angles.

**Methods:**

In this randomized clinical trial study, a total of 103 second-grade male students with FHP were enrolled. Participants were randomly assigned to experimental and control groups. CVA and SA of participants were measured before and after the 8-week selective corrective exercise program (including strengthening and stretching exercises). The photogrammetric method was used to measure CVA and SA. MCID value was calculated for CVA and SA using the distribution method.

**Results:**

The results showed that there was a significant difference between the experimental and control groups in terms of CVA (F = 89.04, *P* = 0.005, Effect size = 0.47) and SA (*F* = 18.83, *P* = 0.005, Effect size = 0.16). After eight weeks of selective corrective exercises, the MCID values of CVA and SA were 1.40° and 1.34°, respectively.

**Conclusion:**

This study revealed that the selective corrective exercises might lead to postural correction of students having FHP problem. Results further indicated that a corrective exercise program would be considered beneficial if it increased CVA and SA values at least 1.40 and 1.34 degrees, respectively.

**Supplementary Information:**

The online version contains supplementary material available at 10.1186/s12887-022-03300-7.

## Introduction

Proper posture is a state of musculoskeletal balance in which a minimum load imposes on the body structures [1]. Despite the possible impact of posture on people's musculoskeletal and psychosocial health [2], many people suffer from poor posture. Forward head posture (FHP), defined as a protrusion of the head in the sagittal plane, is a key indicator of poor posture [3]. This postural abnormality is characterized by flexion of the lower cervical spine (C4-C7) and hyperextension of the upper cervical spine (C1-C3) [4, 5]. It is suggested that FHP is associated with shortened sternocleidomastoids, upper trapezius, levator scapulae, suboccipital muscles, and weakened deep cervical flexor muscles [4, 6].

FHP is one of the most common postural deviations among different populations, and its prevalence has been investigated in several studies [3, 7–10]. The prevalence of FHP is reported to be approximately 60–70% in students in Malaysia and India [8, 10]. Furthermore, it is shown that FHP is a common postural disorder at different educational levels of schools [11], while more than 50% of Iranian students had FHP disorder [7]. These studies conducted in different populations show the high prevalence of this problem and the need for more attention. It seems that several factors, including gender and educational level [11], carrying schoolbag [12], electronic devices usage [13], physical activity behaviors, and psychological aspects [2], may predispose the development of FHP among students.

Previous studies have shown that FHP is associated with numerous musculoskeletal disorders, such as temporomandibular disorder, tension-type headache, shoulder and neck pain, trigger points in the suboccipital muscles, reduced vital capacity, and dyskinesia at the shoulder complex and cervical spine [6, 14–16]. Further consequences of FHP include decreased cervical proprioception, increased reaction times and movement velocity of the Center Of Gravity, subacromial impingement syndrome, thoracic outlet syndrome, and vestibular hypo function [16–20]. It therefore seems that the correction of FHP may play an important role in managing and preventing these consequences [5].

Previous studies have addressed the effects of different exercises and modalities on FHP, including scapular stabilization exercises, Kendall's stretching and strengthening exercises, elastic band exercises, proprioceptive training, cervical stabilization exercises, sensorimotor training, and dynamic neuromuscular stabilization exercise [5, 21, 22]. All studies showed the significant positive effects of different exercises on FHP, but there is a lack of study focusing on the minimal clinically important difference (MCID) in variables related to FHP.

The MCID term was first described by Jaeschke et al. (1989). They suggested that MCID can be expressed as "the smallest difference in score in the domain of interest which patients perceive as beneficial and which would mandate, in the absence of troublesome side effects and excessive cost, a change in the patient's management" [23]. Clinical trials usually report whether their interventions lead to statistically substantial effects or not [24]. A statistically significant result does not necessarily denote the perceived benefits by clients or any clinical relevance [24]. Determining the usefulness of intervention at a minimal level in a clinical trial is crucial. It can serve as a standard for establishing a clinically meaningful effect of an intervention [24, 25]. MCID can be defined as the minimal effect that is meaningful to clients. It is important to know what minimum amount of change in angles, representing FHP, is necessary for clients to feel an actual improvement in their condition.

To the best of our knowledge, researchers have not calculated the exact clinical values for the forward head posture based on the MCID criterion after prescribing corrective exercises. Therefore, we designed an eight-week corrective exercise program, aimed to examine its effect on students having FHP disorder and, to introduce the MCID values for Craniovertebral Angle (CVA) and Shoulder Angle (SA).

## Methods

### Participants

**Fig. 1:**
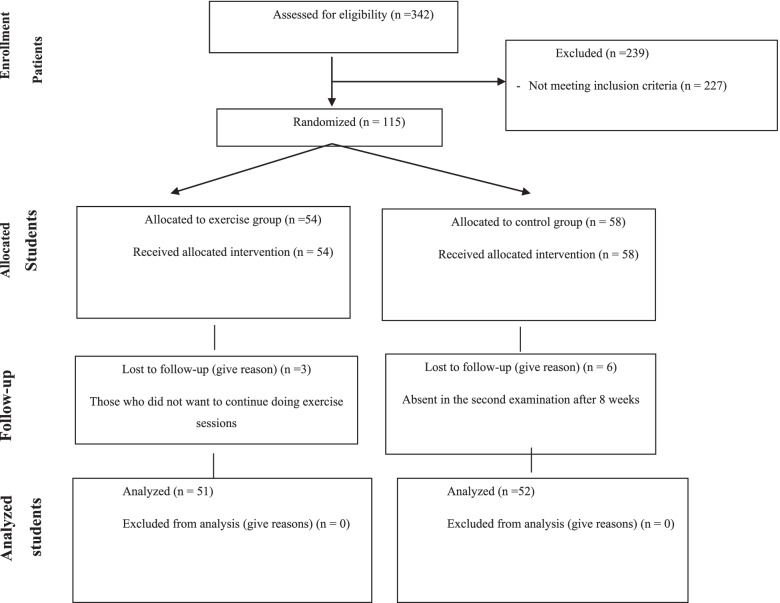
Modified CONSORT flow diagram for individual randomized controlled trials of non-pharmacologic treatments

In this single-blinded randomized clinical trial study, convenience sampling was used to recruit 103 male elementary school students with FHP (Fig. 1). Based on the CVA changes in literature and considering effect size = 0.446, α = 0.05, and power of 80%, the sample size was 49, determined by G ˟ Power software V. 3.1 for running ANCOVA test [21]. As we wanted to be sure that we had enough people at the end of the study, we recruited 112 persons to prevent problems related to dropping out.

The study inclusion criteria were a CVA less than 50° [26], and being a male elementary school student. Participants were excluded if they had a history of heart disease, structural scoliosis, and kyphosis abnormalities based on the New York postural assessment form. Other exclusion criteria included a history of chronic pain in cervical and lumbopelvic areas, a history of any disorders in postural control, the involvement in regular and professional sports activities, severe visual impairments, and any medical limitation to participate in physical training programs. Participants were further excluded if they missed practice for two consecutive sessions or three non-consecutive sessions during the study. The study methodology was approved by the Iranian Registry of Clinical Trials (IRCT) and registered in the IRCT registration number of IRCT20200927048851N1 and registration date of 2020–11-08) (www.irct.ir). All participants were assured that their data will be confidential, and they could leave the study whenever they wanted.

### Procedure

First, during a familiarization session, the process and objectives of the study were explained to participants and their parents. Then, informed written consent was obtained from ' legal guardians of all participants (s). In addition, informed consent obtained from all legal guardians for publication of identifying images in an online open-access publication.

Demographic data, in addition to medical history, was recorded for each participant later. Subjects with CVA less than 50° were randomly (simple randomization method) assigned into experimental (*N* = 51) and control (*N* = 52) groups using a random number table, which is in the form of numbers 0 to 60 for the control group, numbers 61 to 120 for the group of corrective exercises. Randomization was done using a random number table. The participants were asked to close their eyes and move their finger in the predetermined direction to touch one of the numbers. Then, the first author records the number and assigns individuals into the relevant groups based on them. The experimental group performed selective corrective exercises for eight weeks with an interval of three sessions per week. The control group did not participate in any postural correction protocols at the study time. However, they assured that they could participate in the same protocol as the experimental group after the end of the study if they wanted. Before and after eight weeks of selective corrective exercises, the data were collected from both groups using the photogrammetric method. Furthermore, after eight weeks, a blinded photographer took pictures of each student after the last exercise session.

The previous studies have suggested the photogrammetric method as a sensitive and reliable method for postural assessment [27–29]. The exercise protocol was performed under the direct supervision of the first author. All participants who had two consecutive sessions or three non-continuous sessions of absence were excluded from the study. A blinded examiner took photographs and extracted data from photographs.

The CVA and SA angles were obtained using a commercial camera (Canon Camera, D5600). The camera was placed on a tripod holding at a distance of 80 cm apart from the frontal plane to the participants. The camera's height was set at the level of the seventh cervical vertebra for each participant. The privacy and comfort of the participants were guaranteed [30, 31].

Skin markers were placed on the acromion, ear tragus, and spinous process of the seventh cervical vertebra. Three pictures were taken from a lateral view to obtain CVA and SA. At the end, the photos were moved to the computer, and the target angles were calculated using the AutoCAD software (release 33, 2018). The mean average of angles extracted from three photos was considered as CVA and SA measures [32].

**Fig. 2:**
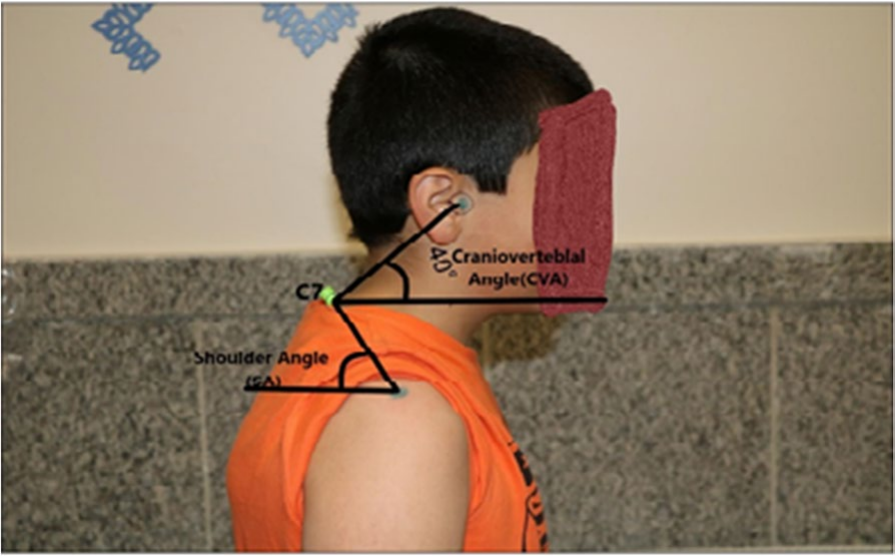
The angles in lateral view-Craniovertebral (CVA), Shoulder (SA)

The angles in the lateral view were measured (Fig. 2) as follows:

- **Craniovertebral angle (CVA):** It was considered as the angle between a horizontal line through the spinous process of the C7 vertebra and a line from the spinous process of the C7 vertebra through the ear tragus [1]. If this angle was less than 50°, the subject were included in the study [33].
- **Shoulder angle (SA):** It was considered as the angle between a horizontal line through the spinous process of the C7 vertebra and a line passes from the midpoint of the shoulder joint through the spinous process of the seventh cervical vertebra [28].

### The selective corrective exercises (SCEs) program

**Fig. 3:**
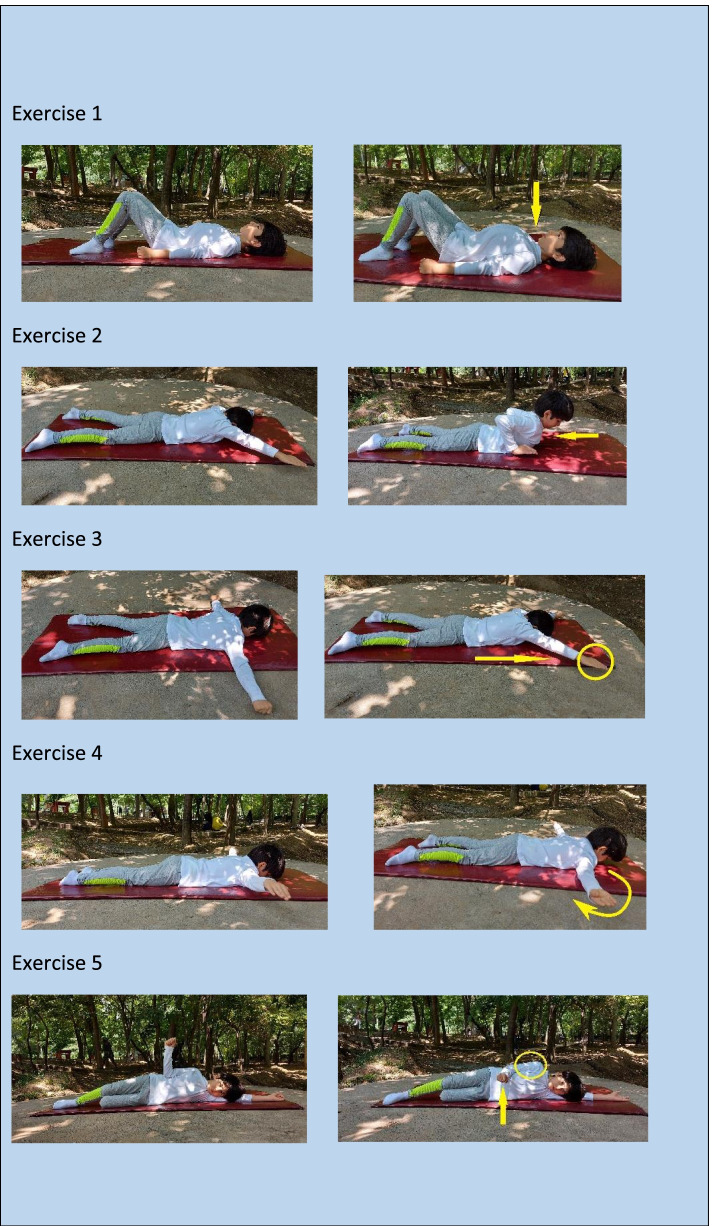
Digital images of a child while performing the strengthening exercises

**Fig. 4:**
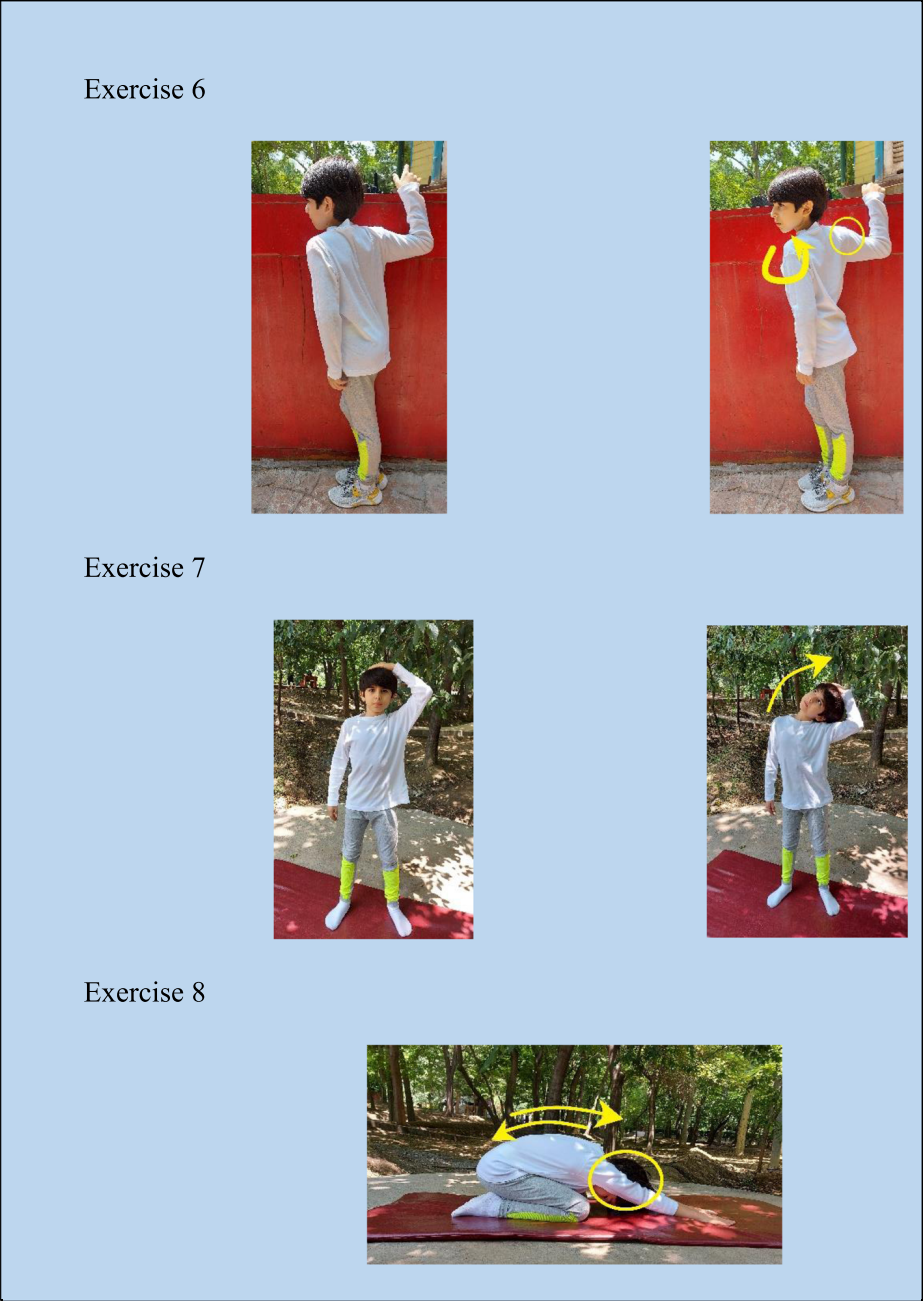
Digital images of a child while performing the stretching exercises

The SCEs program was comprised of four strengthening (targeting longus colli, longus capitis, middle trapezius, lower trapezius, serratus anterior, rhomboids, teres minor and infraspinatus muscles) and three stretching (targeting pectoralis minor, sternocleidomastoid, and levator scapulae muscles) exercises [34]. The strengthening exercises included (a) chin tuck in supine lying with the head in contact with the floor, (b) Y-to-I exercise in a prone position, (c) prone horizontal abduction with external rotation, and (d) side-lying external rotation. The stretching exercises included (a) one-sided unilateral self-stretch of pectoralis minor in standing position, (b) static sternocleidomastoid stretch, and (c) static levator scapulae stretch [34]. The experimental group performed an 8-week exercise program three times per week. The control group was asked not to participate in the corrective exercise program or other regular exercises during this study. However, they were assured that the SCEs would prescribe for them after the study. Participants in the experimental group were trained to complete three sets of 10–15 repetitions of the strengthening exercises and three sets of stretching exercises (each stretching exercise was held for 30–45 s). All exercises were performed at each session of the eight weeks that lasted approximately 30 min. For better supervision, the experimental group was divided into three groups of 15 to 17 people to perform the SCEs in all sessions [35] (Figs. 3–4). All sessions were performed under the direct supervision of the first author who was experienced in Pilates and functional training and had three years of training experience with children.

Training at the *first two* weeks was considered as a familiarization period. In the first two weeks of SCEs, the participants exercised under the supervision of the first author. She taught the exercises by performing them and showing videos to the participants. Then the participants were asked to perform the prescribed exercises truly. In the case of a mistake, the participants were trained to perform the exercises correctly case by case. At the beginning of the third week, one-repetition maximum (1RM) was estimated for participants based on the Brzycki formula ($1RM=w.\left(36\left/ 37-r\right.\right)$) [36] for strengthening exercises. In this formula, *1RM* represents the maximal weight that a person can lift for one repetition, *w* denotes weight lifted in the assessment session, and *r* refers to the number of repetitions that the person completed in the assessment session [36]. To estimate 1RM for strengthening exercises, equal external loads (variable wrist cuff weights with a maximum weight of 500 g) were first added to the bilateral wrists of participants. They were asked to perform every exercise to any number they could do (except for chin tuck in prone). Then the weight of external loads and number of performed repetitions were used to calculate 1RM based on the above formula. From the start of the 3^rd^ week to the end of the 8^th^ week of the SCEs, strengthening exercises were performed with the added weight of 30% of each participant's 1RM as an external mechanical resistance [21]. The weight was added to the participants' bilateral wrists in all strengthening exercises except for chin tuck in prone exercise performed without adding external load in all eight weeks.

During weeks 3-5, the experimental group was asked to perform ten repetitions of strengthening exercises with 30% of 1RM and 30 s of stretching exercises. In weeks 6–8, the participants carried out 15 repetitions of the same strengthening exercises and 45 s of stretching exercises according to Table 1. Some images of a child during strengthening and stretching exercises are presented in Figs. 3 and 4.

**Table 1:** Brief description of various employed exercises (Strengthening and Stretching), their entangled muscles, and the duration or frequency of the exercise during the training program; all exercises were prescribed for three sets for each session

No	Type of Exercise	Entangled Principle Muscle	Class of Exercises	Duration (s) or Frequency of the exercise week [3-5]	Duration (s) or Frequency of the exercise week [6-8]
1	Chin tuck in supine lying with the head in contact with the floor	✓ Longus colli ✓ Longus capitis	Strengthening	10 rep	15 rep
2	Y-to-W exercise in the prone position	✓ Rhomboids ✓ Upper trapezius ✓ Middle trapezius ✓ Posterior deltoid ✓ Rotaror Cuff (Supraspinatus)	Strengthening	10 rep	15 rep
3	T-to-Y exercise in a prone position	✓ Middle trapezius ✓ Lower trapezius ✓ Serratus anterior	Strengthening	10 rep	15 rep
4	Prone horizontal abduction with external rotation	✓ Middle trapezius ✓ Lower trapezius ✓ Rhomboids ✓ Infraspinatus ✓ Teres Minor	Strengthening	10 rep	15 rep
5	Side-lying external rotation	✓ Teres minor infraspinatus	Strengthening	10 rep	15 rep
6	One-sided unilateral self-stretch of pectoralis minor in a standing position	✓ Pectoralis minor	Stretching	30 s	45 s
7	Static sternocleidomastoid & levator scapulae stretch	✓ Sternocleidomastoid ✓ Levator scapulae	Stretching	30 s	45 s
8	Cat stretch	✓ Latissimus dorsi ✓ Middle trapezius ✓ Lower trapezius ✓ Serratus anterior	Stretching	30 s	45 s

### Statistical analysis

The data were analyzed using SPSS Version 25. The normal distribution of all data was examined using the Kolmogorov–Smirnov test, and the homogeneity of variance between groups was assessed using Levene's test. To determine between-group difference, the data were analyzed by analysis of covariance (using ANCOVA and entering the pre-intervention values as covariates in the statistical model) and independent t-tests. Estimated MCID of the CVA and SA was calculated by the distribution method that uses effect size (EZ) obtained from partial eta squared values, standard deviation (SD), and standard error of measurement (SEM) according to this formula $SEM=SD\times \sqrt{1-ICC}$ [37].

## Results

There were nine dropouts in the study (3 in exercise and 6 in control groups), so the remaining data obtained from participants were used in the final data analysis. Participants' demographic characteristics, including age, height, weight, and Body Mass Index (BMI) in both experimental and control groups are presented in Table 2. The independent T-test results demonstrated that there was no significant difference between two groups in terms of demographic characteristics.

**Table 2:** Demographic characteristics of participants in both experimental and control groups at the beginning of the study

Variables	Experimental group (*N* = 51)	Control group (*N* = 52)	t	*P*-value
Age (years)	11.8 ± 0.87^a^	11.94 ± 0.78	-0.85	0.97
Height (cm)	151.00 ± 7.36	150.00 ± 8.82	1.01	0.313
Weight (kg)	49.31 ± 1.17	46.52 ± 1.12	1.24	0.219
BMI(kg/m^2^)	21.13 ± 3.5	20.47 ± 3.8	0.90	0.372

a = Mean ± SD, *BMI *Body Mass Index

**Fig. 5:**
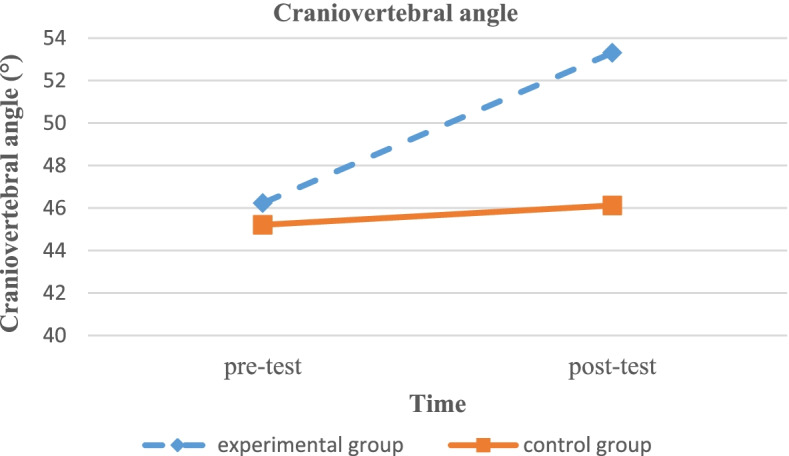
Changes in craniovertebral angles from pre-test to post-test

**Fig. 6:**
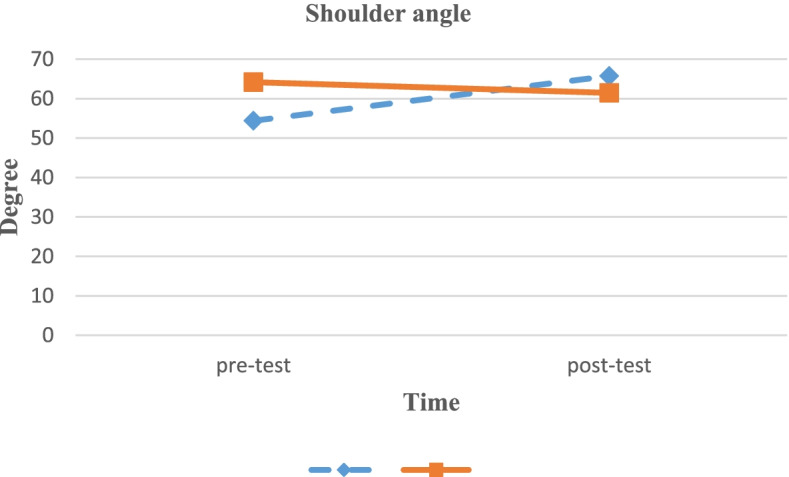
Changes in shoulder angles from pre-test to post-test

The Kolmogorov–Smirnov test results revealed that the data were normally distributed (*p* > 0.05). The ANCOVA was run to analyze the possible effects of SCEs on the study variables by adding pre-intervention values as covariates in the statistical model. The ANCOVA results are summarized in Table 3. The results revealed that a significant difference was observed between the two groups with respect to post-test angles, the CVA and SA (Figs. 5–6).

**Table 3:** ANCOVA results of CVA and SA to compare mean average of pretest and posttest measured angels (°), and evaluate the effect size by eliminating possible effects of pretest measures

Variable	Angel (°) for Experimental group (Mean ± SD)	Angel (°) for Control group (Mean ± SD)	*P*-value	Effect size
CVA (pretest)	46.23 ± 2.93	45.21 ± 3.75	0.005^*^	0.42
CVA (posttest)	53.13 ± 3.43	46.12 ± 4.56		
SA (pretest)	54.37 ± 15.73	64.15 ± 13.41	0.005^*^	0.16
SA (posttest)	65.74 ± 13.30	61.47 ± 13.23		

*CVA *Craniovertebral Angle, *SA* Shoulder Angle, *SD* Standard Deviation, *: Statistically significant differences observed

Then Intraclass Correlation Coefficient (ICC) was calculated for CVA and SA by using three trials of pretest measures in all participants. The MCID values of CVA and SA after eight weeks of SCEs were 1.40° and 1.34°, respectively. More details are presented in Table 4.

**Table 4:** MCID values after 8 weeks of corrective exercises and related data

Variable	ICC (95% CI)	SD	SEM	ES	MCID
**CVA**	0.983(0.976–0.988)	3.88	0.51	0.48	1.40
**SA**	0.999 (0.999–0.999)	15.34	0.49	0.16	1.34

*CVA* Craniovertebral Angle, *SA *Shoulder Angle, *ICC *Intraclass correlation coefficient, *SD *Standard deviation, *SEM *standard error of measurement, *ES *Effect size, *MCID *Minimal clinically important difference

## Discussion

In this study, we investigated the effects of an 8- week selective corrective exercise program on CVA and SA in students with FHP disorder. In addition, the MCID was established for the mentioned angles. The results revealed that the eight weeks of selective corrective exercises substantially increased CVA and SA, suggesting that the corrective exercises had a significant effect on postural correction.

Small CVA imposes a greater load on the extensor muscles and surrounding connective tissues by increasing the external moment arm [38]. The results of the current study showed that CVA was increased after an 8-week corrective exercise program that was in agreement with previous studies [31, 34, 39, 40]. Harman et al. reported that CVA was increased in normal adults after a 10-week home-based exercise program, including two strengthening exercises targeting shoulder retractors and deep cervical flexors and two stretching exercises targeting pectoral and cervical extensor muscles [31]. In another study, Ruivo et al. reported that in addition to physical education classes, the participation in an 8- month posture corrective exercise program could significantly increase CVA [40]. A result obtained from the current study showing an increase in CVA due to participation in the corrective exercise program was consistent with a meta-analysis suggesting that therapeutic exercises might result in significant changes in CVA [5].

Moreover, Lee et al. found that McKenzie exercises, self-stretch exercises, and Kendall exercise could significantly increase CVA [41]. In comparison to the control group, increased CVA in the experimental group might due to restoring agonist/antagonist muscular balance by increasing the lengthening capacity of shortened muscles and shortening capacity of the lengthened ones [21]. It has been indicated that FHP is associated with tightness in levator scapulae, the sternocleidomastoid, the pectoralis minor muscles, and muscle weakness in the middle and lower trapezius, deep cervical flexors, teres major and minor, serratus anterior, and rhomboids [42].Accordingly, the selective corrective exercise protocol was prescribed in this study to address these tissues [34].

Shoulder Angle (SA) indicates shoulder position in relation to the seventh cervical spinous process [43]. The results of the current study showed that SA was increased after an eight-week corrective exercise program, which was in agreement with previous studies [34, 40, 44], including Seidi et al., who reported a reduction in SA in patients with hyperkyphosis after a 12-week intervention [44]. In another study, Ruivo et al. observed that SA was significant increased after a 16-week stretching and resistance training program [34]. This increase in SA may be due to an increase in muscular strength of the scapular muscles [34].

To the best of our knowledge, this is the first study to determine the MCID of CVA and SA in students with FHP. Our estimates for the MCID of CVA and SA were 1.40° and 1.34°, respectively. In other words, the minimum change in CVA and SA should be 1.40° and 1.34°, respectively. FHP patients feel a real change in their situation. In clinical trials, collating the ratio of participants between groups who attain the MCID is more informative than comparisons of average change between groups because a statistically significant difference does not necessarily show a difference perceived as clinically important by the participants [45]. MCID is acquiring interest and significance in medical research and practice [45], but this concept has been ignored in the corrective exercise domain. For the study participants, the MCID values of 1.4° for CVA and 1.34° for SA can be easily used to evaluate an objective improvement. In clinical practice, these values can be utilized as a common language on reports after prescribing corrective exercises in rehabilitation or other health-related professional programs between care providers, patients, and therapists. In general, it can be helpful to examine the effect of corrective exercises.

The changes of CVA and SA angles improved decimal in amount, so it might be related to the duration of exercise. Therefore, the result of improvement in CVA and SA angles can be discussed after increasing the duration of exercises. On the other hand, previous studies showed that there was a significant relation between psychological factors and people's posture. We can add psychological interventions to our exercises intervention to increase the effectiveness of the study [2]. In addition, according to the aim of this study, we focused on exercise protocol instead of training plans to students. Thus, in future studies, researchers can investigate the effectiveness of exercise training to correct neck posture to improve CVA and SA angles.

The current study had several limitations. First, the study only included male students as participants; therefore, the findings could not be generalized to other populations. Second, this study was accomplished to investigate only the effect of an 8-week selective corrective exercise program on CVA and SA, and a follow-up was not performed. It is better to conduct a study to examine the durability of the effects of the corrective exercises on CVA and SA. Third, the readers should keep in mind that the values of MCID achieved in this study might differ while prescribing other medical interventions or other corrective exercise protocols. Therefore, it can be recommended that more studies be conducted to calculate MCIDs for other interventions of exercise protocols. Finally, our study described only the effects of corrective exercises on the static posture of the head and shoulder; therefore, the findings could not be generalized to dynamic posture.

## Conclusion

This study showed that selective corrective exercises could improve posture in students with FHP. In addition, the MCID values for CVA and SA were 1.40° and 1.34°, respectively. The findings of this study may provide helpful information for corrective exercise and rehabilitation specialists.

## Supplementary Information

**Additional file 1.** 

## Data Availability

The raw data and material will be available online after publishing the paper as a supplementary file in the journal.
