# Correction: Establishing minimal clinically important difference for effectiveness of corrective exercises on craniovertebral and shoulder angles among students with forward head posture: a clinical trial study

**DOI:** 10.1186/s12887-022-03605-7

**Published:** 2022-09-28

**Authors:** Zahra Heydari, Rahman Sheikhhoseini, Shahnaz Shahrbanian, Hashem Piri

**Affiliations:** 1 Department of Corrective Exercise & Sport Injury, Faculty of Physical Education and Sport Sciences, Allameh Tabataba’i University, Tehran, Iran; 2 Department of Corrective Exercise & Sport Injury, Faculty of Physical Education and Sport Sciences, Allameh Tabataba’i University, Western Azadi sport complex boulevard, Hakim Highway, Tehran, Iran; 3 Department of Sport Science, Faculty of Humanities, Tarbiat Modares University, Tehran, Iran; 4 Department of Corrective Exercise & Sport Injury, Faculty of Physical Education and Sport Sciences, Allameh Tabataba’i University, Tehran, Iran

**Correction: BMC Pediatr 22, 230 (2022)**

**https://doi.org/10.1186/s12887-022-03300-7**

In the originally published version of this article [1] the PARTICIPANTS NAMES was incorrectly included in the first column in supplementary file. The original article has been corrected.

## Supplementary Information

**Additional file 1.**

## References

[1] Heydari Z, Sheikhhoseini R, Shahrbanian S (2022). Establishing minimal clinically important difference for effectiveness of corrective exercises on craniovertebral and shoulder angles among students with forward head posture: a clinical trial study. BMC Pediatr.

